# Surface Plasmon Resonance Effect in Inverted Perovskite Solar Cells

**DOI:** 10.1002/advs.201500312

**Published:** 2016-01-21

**Authors:** Jin Cui, Cheng Chen, Junbo Han, Kun Cao, Wenjun Zhang, Yan Shen, Mingkui Wang

**Affiliations:** ^1^ Wuhan National Laboratory for OptoelectronicsHuazhong University of Science and Technology1037 Luoyu RoadWuhan430074HubeiP. R. China; ^2^ Wuhan National High Magnetic Field CenterHuazhong University of Science and Technology1037 Luoyu RoadWuhan430074HubeiP. R. China

**Keywords:** hybrid, optical manipulation, perovskite, solar cell, surface plasmon resonance

## Abstract

This work reports on incorporation of spectrally tuned gold/silica (Au/SiO_2_) core/shell nanospheres and nanorods into the inverted perovskite solar cells (PVSC). The band gap of hybrid lead halide iodide (CH_3_NH_3_PbI_3_) can be gradually increased by replacing iodide with increasing amounts of bromide, which can not only offer an appreciate solar radiation window for the surface plasmon resonance effect utilization, but also potentially result in a large open circuit voltage. The introduction of localized surface plasmons in CH_3_NH_3_PbI_2.85_Br_0.15_‐based photovoltaic system, which occur in response to electromagnetic radiation, has shown dramatic enhancement of exciton dissociation. The synchronized improvement in photovoltage and photocurrent leads to an inverted CH_3_NH_3_PbI_2.85_Br_0.15_ planar PVSC device with power conversion efficiency of 13.7%. The spectral response characterization, time resolved photoluminescence, and transient photovoltage decay measurements highlight the efficient and simple method for perovskite devices.

## 1 Introduction

Solar cells using hybrid lead halide perovskite as light harvester have evidenced significant progress in the past few years.^1^, ^2^, ^3^, ^4^ Currently, the certified efficiency for perovskite solar cells (PVSC) is 20.1% and expected to improve further.^5^ This success is closely associated with the photoelectrical properties of the specific light absorber, such as CH_3_NH_3_PbX_3_ (X = I, Br, Cl), exhibiting an appropriate band gap, small exciton binding energy, and long and balanced ambipolar charge transport.^6^, ^7^ Most efficient perovskite devices rely on the p‐i‐n heterojunction structure, in which TiO_2_ and 2,2′,7,7′‐tetrakis (*N*,*N*‐di‐*p*‐methoxyphenylamine)‐9,9′‐spirobifluorene (spiro‐MeOTAD) are the widely used electron and hole transporting materials, owing to their good optical transparency and well band alignment with respect to CH_3_NH_3_PbI_3_.^8^, ^9^ Recently, an emerged inverted PVSC, has attracted intensive interests, in which the photo‐induced holes are collected at the front transparent conductive glass substrate after photoexcitation.^10^ **^–^** 12 Perovskite devices will be certainly benefited from new materials and device configurations, and thus, resolving some obstacles existing in the conventional cells, such as the current–voltage scan hysteresis.^13^, ^14^ Recently, the elimination of photocurrent hysteresis in the inverted CH_3_NH_3_PbI_3_‐based solar cells has been demonstrated by different groups.^15^, ^16^

Poly(3,4‐ethylenedioxythiophene) poly(styrene‐sulfonate) (PEDOT:PSS) has been used as hole selective contact in inverted perovskite cells, achieving outstanding power conversion efficiency (PCE) of about 15%.^17^, ^18^ Meanwhile, NiO‐based PVSCs have also shown growing attention, due to its high stability and electrical conductivity.^19^ **^–^** 21 For example, a faster charge transfer between NiO and charge transfer materials than that of TiO_2_ has been revealed with scanning electrochemical microscopy.^21^ Chen et al. first reported the replacement of PEDOT:PSS with a thin NiO hole selective layer, resulting in a PCE of 7.8%.^11^ We applied a reactive magnetron sputtered NiO ultrathin layer to promote the efficiency up to 9.8%.^22^ Subsequently, Chen et al. proposed a hybrid interfacial layer with an important “dual blocking effect,” resulting in PCE of ≈13.5%.^23^ Recently, CH_3_NH_3_PbI_3_‐based solar cell with 15.4% PCE was demonstrated by using solution‐processed Cu‐doped NiO hole selective interlayer.^24^ The outstanding performance was attributed to an improved electrical conductivity of NiO. An applicability of Cu:NiO*_x_* with large band gap (*E*_g_) perovskite [CH_3_NH_3_PbI_3−*x*_Br*_x_*] solar cell has also been pointed out in this work. The PVSC devices using CH_3_NH_3_PbI_3−*x*_Br*_x_* with large *E*_g_ encounter less potential loss and exhibit high photovoltage of 1.13–1.16 V. However, a relative low photocurrent of 8–12 mA cm^−2^ was obtained due to a less utilization of photons in the wavelength range from 670 to 780 nm. More recently, a remarkable efficiency of 17.3% was achieved on devices using pulsed laser deposited NiO.^25^ The (111)‐oriented nanostructured NiO film with good optical transparency plays a key role in the efficient extraction of holes and the prevention of electron leakage.

Optical manipulation has been successfully explored to improve light utilization efficiency in various photovoltaic devices, such as using folded device architecture, aperiodic dielectric stack, diffraction grating, and plasmon resonant metallic nanostructure.^26^ **^–^** 29 Among them, the introduction of metallic nanostructures as localized surface plasmon resonance (LPSR) into photovoltaic devices has been regarded as the most efficient and simple approach.^30^ Actually incorporation of Au@SiO_2_ core–shell nanoparticles into PVSCs has first reported in 2013.^31^ The authors showed the evidence for the reduced exciton binding energy in the perovskite absorber through photoluminescence study. It is known that the LSPR properties originate from collective oscillation of their electrons in response to optical excitation. However, in the previous report the photon absorption of metallic nanostructures can be negligible when considering the broad visible absorption spectrum and high adsorption coefficient of CH_3_NH_3_PbI_3_ active layer in devices.^31^ Therefore, the application of LSPR effect in perovskite devices has been long underestimated in this community. In line with this, the solar cells need to be structured so that light remains trapped inside to increase the photocurrent generation.

In this work, we report on an inverted CH_3_NH_3_PbI_3−*x*_Br*_x_*‐based PVSC employing a hybrid interfacial layer of “compact NiO*_x_*/meso‐Al_2_O_3_” in combination with nanostructured Au nanoparticles. The CH_3_NH_3_PbI_3−*x*_Br*_x_* absorbers with valence band (VB) ≈5.4 eV are specially selected to provide an optimized optical utilization, and well energy alignment with that of NiO to minimize energy loss. Most importantly, the optical band gap *E*_g_ of CH_3_NH_3_PbI_3−*x*_Br*_x_* can be intentionally tuned wide enough, leaving a suitable spectrum window for the Au nanostructures. Therefore, an expected LSPR excitation in the range of 740–860 nm can be promoted by the resonant interaction between electromagnetic field of incident photons and surface charge oscillation of the Au nanorods (NRs). It should be noted that in the wavelength range, the CH_3_NH_3_PbI_3−*x*_Br*_x_* active layer does not absorb any photons. Therefore, we could intentionally investigate SPR effect for efficiency improvement of inverted PVSCs using Au nanoparticles. By imbedding a thin layer of Au NRs in the active layer of the PVSCs, the PCE was improved by a maximum value of 13.7%, which is attributed to enhanced local electric field originate from SPR effect.

## 2 Results and Discussion

**Figure 1:**
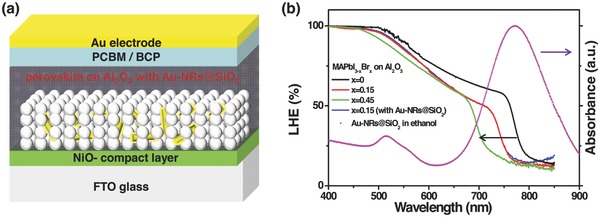
a) Illustration of device structure labeled with different components. b) Light harvesting efficiency (LHE) of CH_3_NH_3_PbI_3−*x*_Br*_x_* absorber (with different Br content) deposited on the same thickness of compact‐NiO/meso‐Al_2_O_3_ layer and UV–visible spectroscopy in ethanol for Au@SiO_2_ NRs with AR of 3.8. The LHE of CH_3_NH_3_PbI_3−*x*_Br*_x_* coated compact‐NiO/meso‐Al_2_O_3_ layer with Au@SiO_2_ NRs is also presented.

**Figure** **1**a depicts the typical inverted PVSC device configuration in this study. To incorporate metal nanoparticles into the perovskite active layer, ethanol solution containing Au@SiO_2_ was added to the Al_2_O_3_ colloid solution at a range of concentrations prior to porous alumina film deposition. The Au@SiO_2_ core–shell metal–dielectric nanoparticles were prepared with a three‐step synthesis process described elsewhere.^32^ X‐ray diffraction (XRD) analysis in Figure S1 (Supporting Information) exhibits a common pattern of bulk gold as well as a broad peak at around 25° corresponding to amorphous silica. Au@SiO_2_ nanoparticles with different AR can be obtained by adjusting either Au seeds or the content of the growth solution.^33^ The corresponding absorption characterization of these Au nanostructures is shown in Figure S2 (Supporting Information). Herein, Au@SiO_2_ nanorods with AR of 3.8 were chosen on account of its most red‐shift absorption peak (≈785 nm). As depicted in Figure 1b, the designed optical manipulation will take full advantage of the incident photons when CH_3_NH_3_PbI_3−_ *_x_*Br*_x_* absorber and Au@SiO_2_ NRs are assembled in such a device. As most of photons in the overlap region could be cut off by light absorbers, a CH_3_NH_3_PbI_3−_ *_x_*Br*_x_* perovskite with onset of absorption band from 786 nm (1.58 eV) to 704 nm (1.78 eV) has been synthesized and tested in this study. The light harvesting in the Au@SiO_2_ device is indistinguishable from the one without Au@SiO_2_ nanostructures due to a low loading of Au@SiO_2_ NRs (2.0 wt%) in the Al_2_O_3_ film.

**Figure 2:**
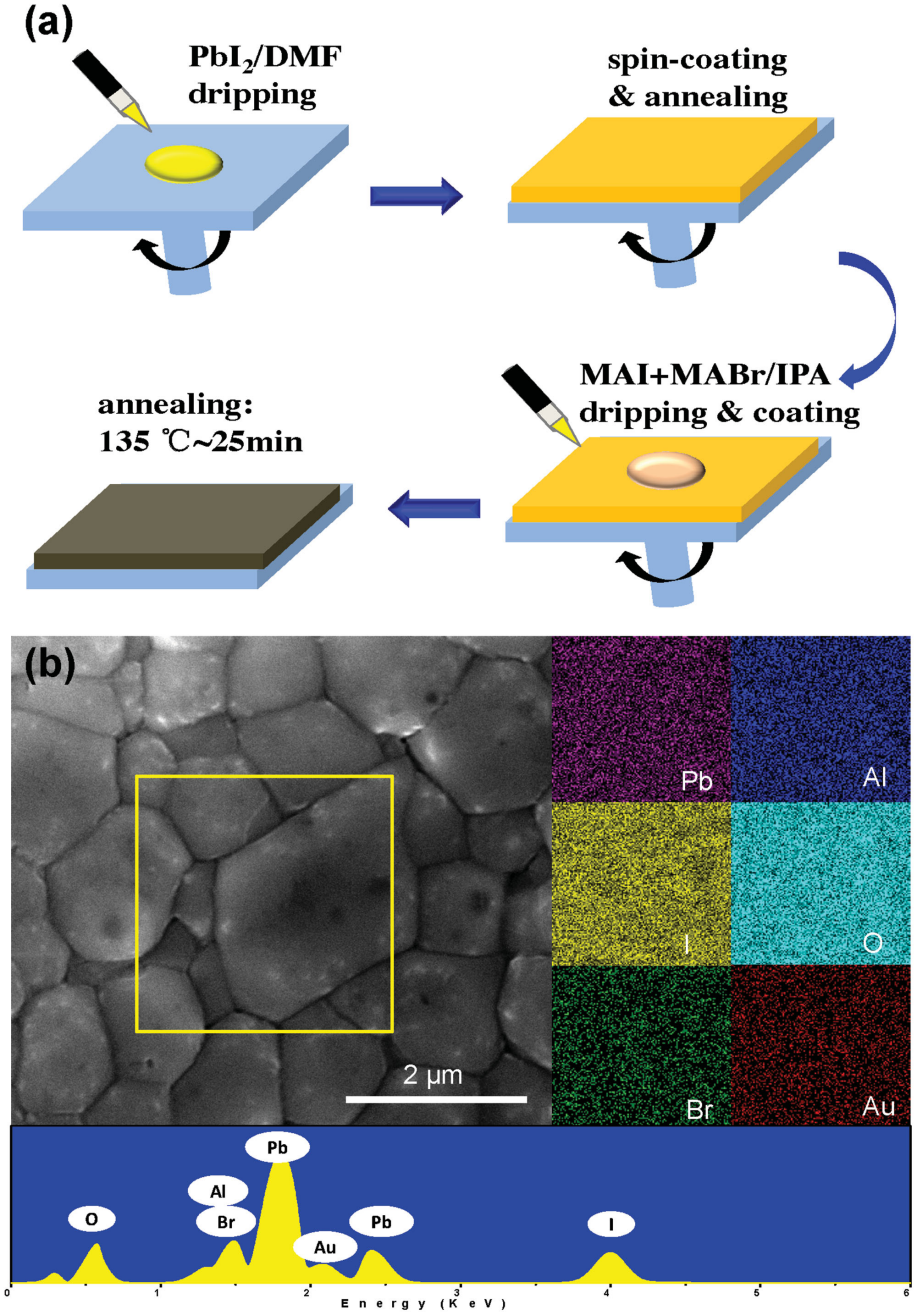
a) Schematics of the preparation approach to CH_3_NH_3_PbI_3−*x*_Br*_x_* perovskite film. b) Top view SEM image of CH_3_NH_3_PbI_3−*x*_Br*_x_* coated meso‐Al_2_O_3_ film with Au‐NRs and EDX mapping results of the yellow square region.

The CH_3_NH_3_PbI_3−_ *_x_*Br*_x_* absorber was deposited by spun stacking of double layers of PbI_2_ and CH_3_NH_3_I+CH_3_NH_3_Br as illustrated in **Figure** **2**a. Details of the perovskite film preparation procedure can be found in the Experimental Section. Figure 2b shows the scanning electron microscopy (SEM) surface image of as‐prepared CH_3_NH_3_PbI_3−_ *_x_*Br*_x_* film on the compact‐NiO/meso‐Al_2_O_3_ layer. The grain size increases fast to ≈1 μm in a 25 min annealing process. It has been reported that the big grain size CH_3_NH_3_PbI_3−_ *_x_*Br*_x_* has less inner grain boundaries, which is benefited for reduction of charge recombination.^34^, ^35^ The EDX mapping result shows that “Au,” “Br,” and “I” elements are homogeneously distributed in the meso‐Al_2_O_3_ layer (Figure 2b), reflecting a good evidence of the existence of Au NPs and CH_3_NH_3_PbI_3−_ *_x_*Br*_x_* materials.

**Figure 3:**
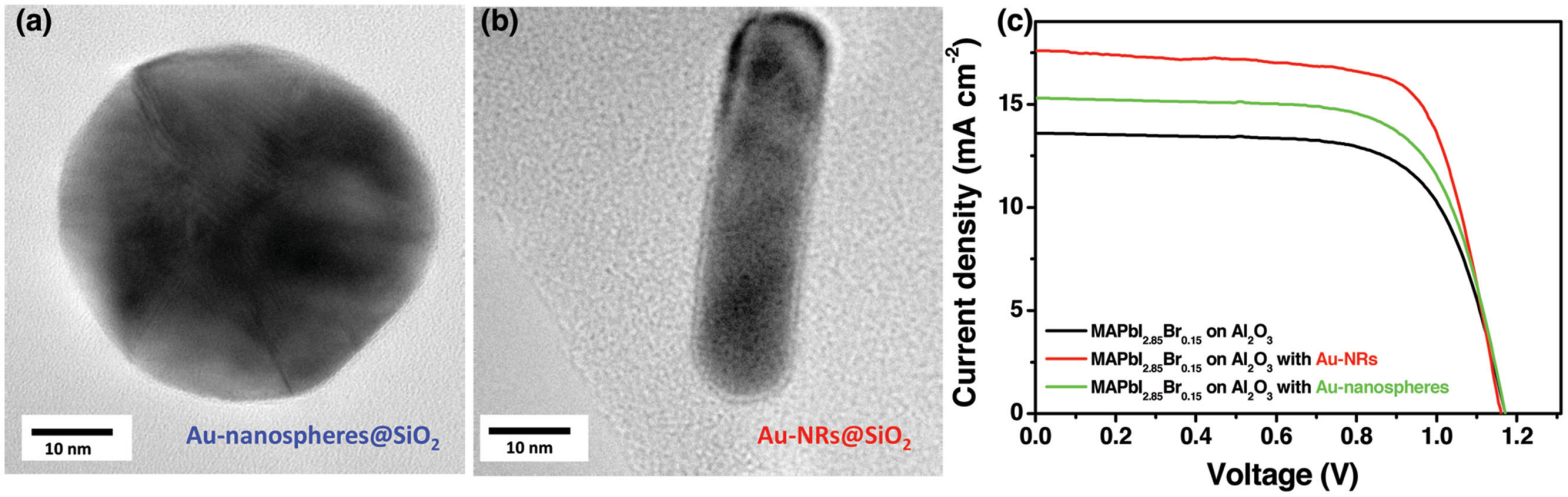
TEM images of the synthesized core–shell SiO_2_ coated a) Au‐nanospheres@SiO_2_ (AR = 1) and b) Au@SiO_2_ NRs (AR = 3.8). c) Photocurrent–voltage characterization of CH_3_NH_3_PbI_2.85_B_0.15_‐based PVSCs with and without Au nanostructures.

The evaluation of validity and rationality of our strategy on optical manipulation was first carried out with consideration of (1) Au NRs with various AR values, and (2) CH_3_NH_3_PbI_3−*x*_Br*_x_* with different Br contents. The absorption spectra of CH_3_NH_3_PbI_3−_ *_x_*Br*_x_* absorber can be controlled through tuning CH_3_NH_3_I/CH_3_NH_3_Br molar ratio during the two‐step spin‐coating process. The used Au nanospheres with diameter of 41 nm (AR = 1, **Figure** **3**a) and Au‐NRs of about 42 nm length and 11 nm width (AR = 3.8, Figure 3b) were coated with an approximate 1–2 nm SiO_2_ shell. The absorption peaks are observed at ≈541 and ≈785 nm for the two Au nanostructure samples in ethanol (Figure S2, Supporting Information), respectively. After adding Au‐nanosphere (AR = 1) into the meso‐Al_2_O_3_ film, the photocurrent was increased by 12% from 13.6 to 15.3 mA cm^−2^ for the CH_3_NH_3_PbI_3−_ *_x_*Br*_x_* (*x* = 0.15)‐based perovskite device. Keeping the same concentration of the Au additives, the device with Au‐NRs (AR = 3.8) showed even higher photocurrent of 17.5 mA cm^−2^ (Figure 3c). Compared to that of Au‐NRs, the *J*_sc_ enhancement of perovskite devices with Au‐nanospheres has been suppressed, mainly due to an optical trapping competition raised from high extinction coefficient CH_3_NH_3_PbI_3−_ *_x_*Br*_x_* (*x* = 0.15) absorber around 541 nm (Figure 1b).

The photovoltaic performance for devices using compact‐NiO/meso‐Al_2_O_3_ with/without Au@SiO_2_ NRs (AR = 3.8)/CH_3_NH_3_PbI_3−_ *_x_*Br*_x_*/[6,6]‐phenyl C_61_‐butyric acid methyl ester (PCBM)/bathocuproine (BCP)/Au configuration by varying Br content in perovskite was further investigated (Figure S3, Supporting Information). Indeed, as expected, the perovskite devices in presence of Br component exhibited higher *V*_OC_ than the pristine CH_3_NH_3_PbI_3_‐based counterparts (Figure S3b, Supporting Information), confirming superior energy level alignment between CH_3_NH_3_PbI_3−_ *_x_*Br*_x_* and NiO. The photocurrent of PVSC devices with adding Au@SiO_2_ NRs was higher than the control devices (Figure S3a, Supporting Information), which originates from the reutilization of photon in longer wavelength region around 785 nm. Increasing the Br portion in the CH_3_NH_3_PbI_3−_ *_x_*Br*_x_* decreases the device's photocurrent. The decrease of photocurrent in the Br portion of *x* = 0–0.45 was accompanied by an augmentation of the *V*_OC_ compensating the decrease in the fill factor. As a result, the overall power conversion efficiency reaches a maximum value of 13.5% at *x* = 0.15 over 15 piece of devices. The statistical data for device performance are shown in Figure S3a (Supporting Information). Therefore, the CH_3_NH_3_PbI_2.85_Br_0.15_ absorber in combination with Au@SiO_2_ NRs (AR = 3.8) takes the most advantage of the optical manipulation design. The work function analysis by photoelectron spectroscopy for the spin‐coated CH_3_NH_3_PbI_3−_ *_x_*Br*_x_* perovskite film in line with the cell energy levels are depicted in Figure S4 (Supporting Information).

**Figure 4:**
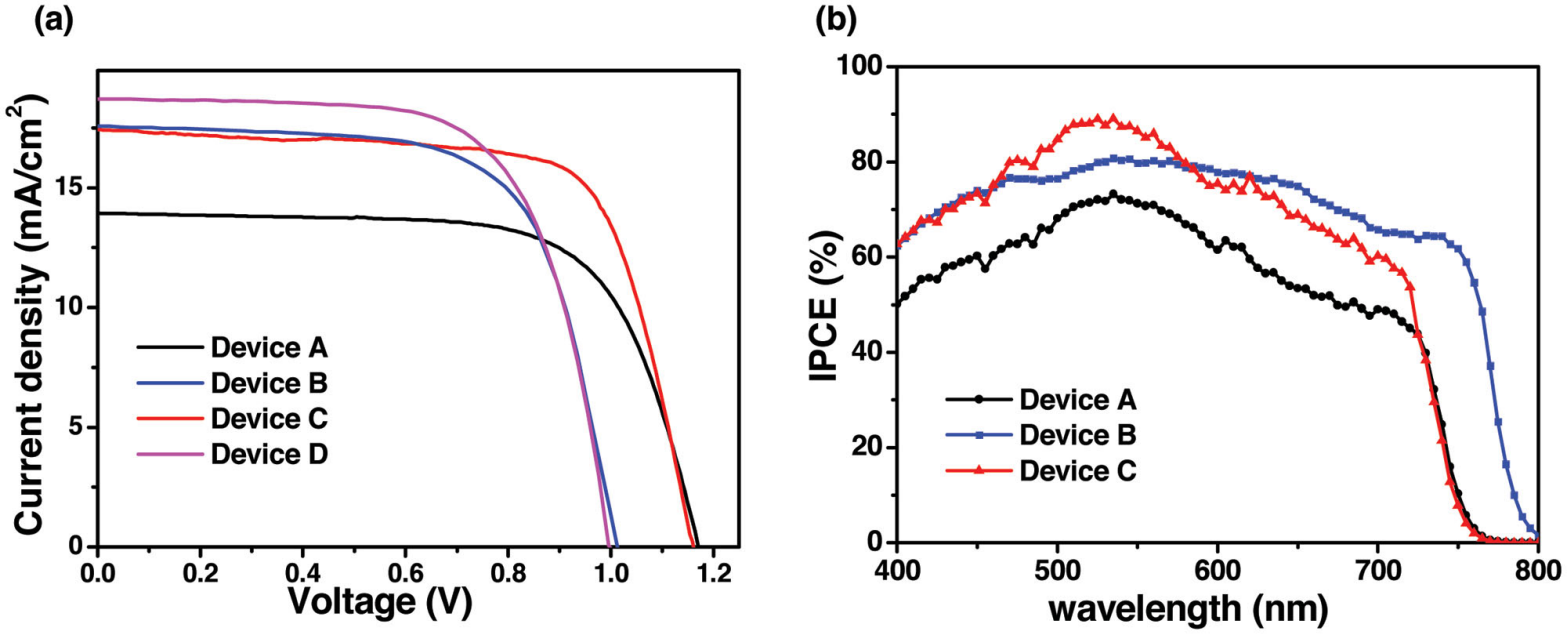
a) Representative *J*–*V* curves for devices using Al_2_O_3_‐only (devices A and B) and Al_2_O_3_ film incorporated with Au@SiO_2_ NRs (devices C and D) with MAPbI_2.85_Br_0.15_ (devices A and C) or MAPbI_3_ (devices B and D) absorbers measured under AM1.5 simulated sunlight (100 mW cm^−2^ irradiance). b) IPCE spectra of corresponding devices. MA: CH_3_NH_3_^+^ cation.

Some photovoltaic experiments were conducted to evaluate the performance of devices by varying the concentration of Au nanoparticles. It was found that the Au NRs concentration played a vital role on the devices performance. Figure S5 (Supporting Information) presents the photovoltaic parameters for the CH_3_NH_3_PbI_2.85_Br_0.15_‐based perovskite device as the concentration of Au@SiO_2_ NRs (AR = 3.8) varies from 0 to 4.2 wt%. The introduction of Au@SiO_2_ NRs results in an increased photocurrent, reaching a maximum value of 17.4 mA cm^−2^ at 2.0 wt% of Au@SiO_2_ NRs. The *V*_OC_ is remarkably kept similar for various devices (Figure S5b, Supporting Information). **Figure** **4**a compares the photocurrent density–voltage (*J*–*V*) curves of the CH_3_NH_3_PbI_3_ or CH_3_NH_3_PbI_2.85_Br_0.15_ based PVSC devices W/O 2.0 wt% incorporation of Au@SiO_2_ NRs within the same thickness meso‐Al_2_O_3_ film. The photovoltaic parameters are tabulated in **Table** **1**. Device A (with Al_2_O_3_‐only using CH_3_NH_3_PbI_2.85_Br_0.15_) exhibits the PCE of 10.7% with a *J*_SC_ of 13.9 mA cm^−2^, a *V*_OC_ of 1.17 V, and a fill factor of 0.66. The replacement of CH_3_NH_3_PbI_2.85_Br_0.15_ with CH_3_NH_3_PbI_3_ results in a significant increase in *J*_SC_, achieving a PCE of 11.3% for device B. The addition of Au@SiO_2_ NRs in devices C and D result in a significant increase in *J*_SC_. Device C with compact‐NiO/meso‐Al_2_O_3_ with Au@SiO_2_ NRs/CH_3_NH_3_PbI_2.85_Br_0.15_/PCBM/BCP/Au configuration showed the highest PCE of 13.7% with a *V*_OC_ of 1.16 V, a *J*_SC_ of 17.4 mA cm^−2^, and a fill factor of 0.68. Devices A and C show higher *V*_OC_ s compared to devices B and D, indicating CH_3_NH_3_PbI_3−_ *_x_*Br*_x_* has better energy alignment with the deep VB of NiO. Considering the similar configuration in the four devices, the augmented output photovoltage and current could be contributed to the synergy effect from Au@SiO_2_ NRs and CH_3_NH_3_PbI_2.85_Br_0.15_ absorber. Figure 4b presents the incident photon‐to‐current conversion efficiency (IPCE) for the corresponding devices. Even though the difference of light harvesting capability can be ignorable for the CH_3_NH_3_PbI_2.85_Br_0.15_‐based devices with and without Au NRs (Figure 1b), an enhanced photocurrent was unambiguously observed for the former. We conclude that the photocurrent improvement is caused by the LSPR effect of Au@SiO_2_ NRs, rather than the scattering effect from metal nanoparticles.

**Table 1:** Photovoltaic parameters of the compact‐NiO/meso‐Al_2_O_3_/CH_3_NH_3_PbI_3−_ *_x_*Br*_x_* (*x* = 0 and *x* = 0.15)/PCBM/BCP/Au configuration inverted PVSCs, with/without 2.0 wt% incorporation of Au@SiO_2_ NPs

Device architecture		*V*_oc_ [V]	*J*_sc_ [mA cm^−2^]	FF	PCE [%]
Device A	CH_3_NH_3_PbI_2.85_Br_0.15_ on Al_2_O_3_	1.17	13.9	0.66	10.7
Device B	CH_3_NH_3_PbI_3_ on Al_2_O_3_	1.01	17.2	0.65	11.3
Device C	CH_3_NH_3_PbI_2.85_Br_0.15_ on Al_2_O_3_ with Au@SiO_2_ NRs	1.16	17.4	0.68	13.7
Device D	CH_3_NH_3_PbI_3_ on Al_2_O_3_ with Au@SiO_2_ NRs	0.99	18.7	0.66	12.2
Device E	CH_3_NH_3_PbI_2.85_Br_0.15_ on Al_2_O_3_ with Au@SiO_2_ nanospheres	1.16	15.3	0.65	11.5

**Figure 5:**
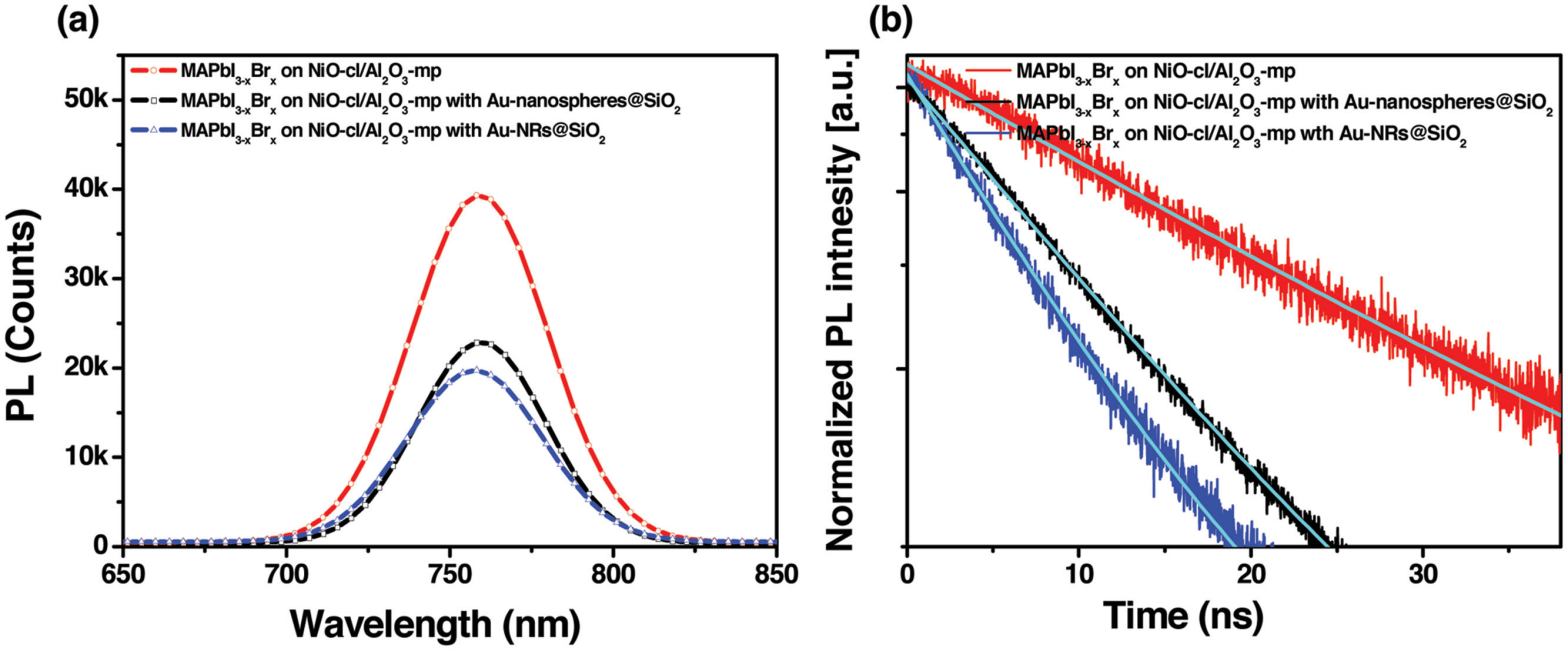
Photoluminescence study: a) Time‐integrated spectra and b) time‐resolved PL decays (detected at 760 nm) for MAPbI_3−*x*_Br*_x_* perovskite coated on meso‐Al_2_O_3_ film with and without incorporating Au‐nanospheres@SiO_2_ (AR = 1) or Au@SiO_2_ NRs (AR = 3.8).

Photoluminescence (PL) measurements on CH_3_NH_3_PbI_2.85_Br_0.15_ coated NiO/meso‐Al_2_O_3_ layer with and without the addition of Au @SiO_2_ nanoparticles (AR = 1 and 3.8) are performed to further probe the influence of the LSPR effect. The excitation wavelength was set at 435 nm in order to avoid any external electromagnetic radiation on Au nanoparticles (absorption peak at 530 nm for that with AR of 1, and absorption peak of 785 nm with AR of 3.8). **Figure** **5**a shows time‐integrated PL spectrum at room temperature, revealing a significant reduction in the signal for the samples incorporating Au NRs over six samples. Figure 5b shows the result of time‐resolved PL measurement at the emission peak (760 nm). An accelerated PL quenching was observed for the sample with the Au NRs. The time constant was evaluated to be 10.9 and 12.1 ns for the sample with Au nanoparticles of AR = 3.8 and AR = 1, respectively, indicating both of them play similar role during the charge separation process. A longer time constant of 24.7 ns was observed for the film without Au nanostructures. The enhanced PL quenching could be contributed from ionization of the excitons and enhanced charge separation, which is related to the LSPR effect of Au NRs.^36^, ^37^

**Figure 6:**
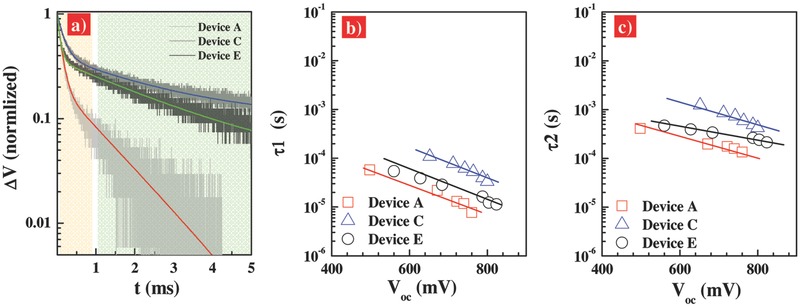
a) Transient photovoltage decay (TPD) data for device A, C in Table 1 and device E with compact‐NiO/meso‐Al_2_O_3_/CH_3_NH_3_PbI_2.85_Br_0.15_/PCBM/BCP/Au configuration with 2.0 wt% incorporation of Au‐nanospheres@SiO_2_, measured under 0.5% sun illumination. And charge recombination lifetime, b) *τ*1 and c) *τ*2, as a function of the open‐circuit voltage, derived from the double exponential fitting of TPD curves.

The interfacial charge‐recombination in the CH_3_NH_3_PbI_2.85_B_0.15_‐based PVSCs was further investigated with transient photovoltage decay (TPD) measurements, which is presented in **Figure** **6**a. This technique is widely employed for the determination of charge lifetimes (*τ_n_*) in sensitized solar cells.^38^, ^39^ Figure S6 (Supporting Information) depicts the typical voltage transient dynamics for all devices responding to the light perturbation. The recombination kinetics in these inverted PVSCs are yet to be fully understood, however, we observed biphasic decays with double exponential functions (Figure 6a). The two time constants (*τ*1 and *τ*2) are suggestive of the presence of two distinguished populations of generated carriers and their recombination independently. Figure 6b,c presents the extracted charge lifetime by fitting photovoltage decay results as a function of the open‐circuit voltage for various devices. This result is different with that observed for dye‐sensitized solar cells.^40^, ^41^ The lifetime constant in the range of 0.1–10 ms depending on the sensitizers and electrolytes can be ascribed to the interfacial charge recombination between electrons in the dye‐covered TiO_2_ and holes in the redox mediators. Therefore, for the perovskite devices, the charge population bearing a short lifetime (*τ*1, in the range of microseconds in Figure 6b) might be attributable to the charge confined within the perovskite bulk layer recombining with defect traps or PCBM. And we associate the longer voltage decay component (*τ*2, in the range from 0.1 to 10 ms in Figure 6c) to the electrons recombining with holes in NiO near the perovskite/charge selective layer interface. An increased lifetime was observed when Au nanoparticles were used. This is contrast to the findings of LSPR effect in dye‐sensitized solar cell. A shorter interfacial charge recombination lifetime of about milliseconds was usually observed for the dye‐sensitized devices when the metallic nanostructures were incorporated.^41^ This observation was explained by a triggered interfacial charge recombination between the metallic nanoparticles and their neighbor mediators.^42^ Previous investigation on dye‐sensitized solar cells revealed that the augmented photocurrent could be contributed both from the scatter effect of metallic nanoparticles and/or the boosted charge separation efficiency when gold NRs was used.^42^ As shown in Table 1, there is about 3.5 mA cm^−2^ enhancement in the *J*_SC_ for device C compared with device A. The charge lifetime for device A is about ten times longer than that of device C. Therefore, this result indicates that the augment in *J*_SC_ for device A could be contributed to the longer lifetime. It is noted that, herein, the perovskite devices with Au @SiO_2_ NRs (AR = 3.8) show the longer charge lifetime than that of devices with Au nanosphere (AR = 1). A long charge lifetime guarantees effective charge collection efficiency, thus the device output photocurrent. This result indicates the aspect ratio of Au nanoparticles could play critical role in the photo‐induced enhanced dissociation of excitons. The similar PL quenching of the films with Au nanostructures with various ARs (Figure 5) indicates the same charge generation rate, however, the device C (with Au NRs) shows an order of magnitude higher in charge lifetime than the device E (with Au nanospheres).

## 3 Conclusion

In summary, we demonstrated the incorporation of Au@SiO_2_ nanorod into the inverted CH_3_NH_3_PbI_2.85_Br_0.15_ perovskite solar cells could significantly increase the device photocurrent, which could be contributed to the LSPR excitation in the range of 740–860 nm by the resonant interaction. An increased interfacial charge recombination lifetime in line with effective charge collection was further observed in the inverted CH_3_NH_3_PbI_2.85_Br_0.15_ perovskite solar cells containing Au nanorods. As a result, a considerably higher PCE of up to 13.7% was achieved. This optical manipulation strategy can be extendable to a broad community of thin film solar cells.

## 4 Experimental Section

*Materials and Sample Preparation*: All solvents and reagents, unless otherwise stated, were of analytically pure quality and used as received. PbI_2_ (99%), Al_2_O_3_ nanoparticles dispersion in isopropanol (<50 nm, 20 wt%), nickel acetylacetonate (95%), super dehydrated solvents of dimethyl formamide (DMF), isopropanol, and chlorobenzene were all purchased from Sigma Aldrich. Synthesis process of Au@SiO_2_ NPs can be referred to the Supporting Information.

*Fabrication of CH_3_NH_3_PbI_3−x_Br_x_‐Based Perovskite Solar Cells*: The ethylammonium lead iodide (CH_3_NH_3_PbI_3_) was prepared according to a previous work.^22^ CH_3_NH_3_PbI_3−_ *_x_*Br*_x_* was deposited with a modified two‐step spin‐coating method.^13^ First, the p‐type selective interlayer, NiO*_x_* was deposited onto the precleaned and prepatterned transparent glass/fluorine‐doped tin oxide (FTO) coated glasses (Pilkington TEC 15 Ω ^−1^) with a spray pyrolysis method. For transparent meso‐Al_2_O_3_ film preparation, 2.0 g Al_2_O_3_ nanoparticles dispersion in isopropanol (20 wt%) was added to 12.0 g α‐terpinol and 2.0 g ethanol solution of ethyl cellulose (10 wt%), the mixture was then treated in ultrasonic bath for 30 min and string for another 30 min to form a homogenous paste. 5 wt% Au@SiO_2_ NRs ethanol solution were mixed with different concentrations (from 0 to 4.2 wt% of Au@SiO_2_ NRs/Al_2_O_3_). The paste was subsequently spin‐coated and sintered at 550 °C for 30 min. A concentration of 460 mg mL^−1^ of PbI_2_ in DMF solution was then spin‐coated onto NiO/Al_2_O_3_ layer (3000 rpm for 30 s). MAI and MABr (MA: CH_3_NH_3_^+^) were mixed with different MAI/MABr molar ratio in 2‐propanol at 0.3 m and then spin‐coated on dry PbI_2_ layer at room temperature. The formation of continuous, compact CH_3_NH_3_PbI_3−_ *_x_*Br*_x_* perovskite films will be completed after annealing at 100 °C for 25 min. The surface morphology of CH_3_NH_3_PbI_3−_ *_x_*Br*_x_* film was characterized by the cross section images of SEM. The PCBM (≈60 nm) and BCP (<10 nm) were spin‐coated inside an argon‐filled glove box. Au layer (65 nm) was thermally deposited on the substrate inside a vacuum chamber (10^−6^ Torr). The active area of the device is 0.16 cm^2^.

*Characterization*: A xenon light source solar simulator (450 W, Oriel, model 9119) with AM 1.5G filter (Oriel, model 91192) was used to give an irradiance of 100 mW cm^−2^ at the surface of the solar cells. Various irradiance intensities from 0.01 to 1.0 sun can be provided with neutral wire mesh 50 attenuators, and the light intensity was calibrated with a standard silicon solar cell. The current–voltage characteristics of the devices under these conditions were obtained by applying external potential bias to the devices and measuring the generated photocurrent with a Keithley model 2400 digital source meter. The *J*–*V* characteristics were recorded by reverse scan or forward scan with a scan rate of 50 mV s^−1^. A similar data acquisition system was used to control the IPCE measurement. SEM images were obtained using FEI Nova NanoSEM 450. XRD results were acquired using Phillips X'Pert PRO. The time‐resolved and steady‐state PL measurements were recorded with Edinburgh instruments (FLSP920 spectrometers). The excitation light source was a femtosecond laser centered at 435 nm, operated at a power of 10 mW.

Transient photovoltage decay measurements were performed on all the cells using a ring of red LED (Lumiled) controlled by a fast solid‐state switch. The pulse widths were 2 ms. An array of InGaN diodes (Lumiled) supplied the white bias light, transients were measured at different white light intensities via tuning the voltage applied to the bias diodes. The voltage output was recorded on an oscilloscope directly connected with the cells.

## Supporting information

As a service to our authors and readers, this journal provides supporting information supplied by the authors. Such materials are peer reviewed and may be re‐organized for online delivery, but are not copy‐edited or typeset. Technical support issues arising from supporting information (other than missing files) should be addressed to the authors.

Supplementary
