# Supplementary material for: Surface Plasmon Resonance Effect in Inverted Perovskite Solar Cells
Source: Adv Sci (Weinh). 2016 Jan 21;3(3):1500312. doi: 10.1002/advs.201500312 (PMC5295766; doi:10.1002/advs.201500312)
Supplement: Supplementary file 1 — Supplementary [file ADVS-3-1500312-s001.pdf]

Copyright WILEY-VCH Verlag GmbH & Co. KGaA, 69469 Weinheim, Germany, 2016.

ADVANCED SCIENCE
Open Access

Supporting Information

for *Adv. Sci.*, DOI: 10.1002/advs. 201500312

Surface Plasmon Resonance Effect in Inverted Perovskite Solar Cells

*Jin Cui, Cheng Chen, Junbo Han, Kun Cao, Wenjun Zhang, Yan Shen, and Mingkui Wang**

Copyright WILEY-VCH Verlag GmbH & Co. KGaA, 69469 Weinheim, Germany, 2013.

Supporting Information

Surface Plasmon Resonance Effect in Inverted Perovskite Solar Cells

*Jin Cui, Cheng Chen, Junbo Han, Kun Cao, Wenjun Zhang, Yan Shen, and Mingkui Wang**

Synthesis and characterization of Au @SiO_2 nanoparticles

Au NRs were fabricated based on a CTAB-assisted seed-mediated growth method reported by Nikoobakht *et al*. with slight modifications.[1, 2] At the Au-seeds preparation procedure, 0.729 g cetyltrimethylammonium bromide (CTAB) and 0.05 mL of 100 mM $HAuCl_4$ were dissolved in 50 mL of Milli-Q water and stirred strongly for 30 minutes in a water bath maintained at 40 ^oC. After the solution cooling down to room temperature, 0.06 mL sodium borohydride ($NaBH_4$) (100 mM, 4 ^oC) was added. The mixture was vigorously stirred for another 5 minutes. The solution changed into dark brown, the flask was steadily kept for 3 hours. To prepare the growth solution, 50 mL CTAB (100 mM, 1.8225 g), 0.25 mL $HAuCl_4$ (100 mM), and 0.5 mL $AgNO_3$ (10 mM) were mixed together, and stirred for 15 minutes at 30 ^oC. After the solution was mixed, 0.2 mL HCl (1 M) and 0.35 mL ascorbic acid (78.8 mM) were added directly and stirred for 3 minutes. Then, 60 uL of the Au-seeds brown solution was injected. The growth procedure need a 5 minutes vigorously stirring and set aside for 20 hours at 30 ^oC. The Au-NRs were collected with centrifugal separation at a speed of 8000 rpm for 20 minutes for 3 times and re-dissolved in deionized water. In this study, the content of $AgNO_3$ was adjusted to control the shape of Au-NRs.

Prior to formation of the silica shell, the purified Au-NRs and 10 uL Tetraethylorthosilicate (TEOS) were dissolved in 4 mL and 590 uL deionized water respectively, with ultrasonic dispersion. The condensation of SiO_2 shells onto the Au-NRs core surface was accomplished by addition of 50 μL of 100 mM NaOH into the dispersion of Au and TEOS mixtures to maintain a final pH value of 10. The thickness of SiO_2 shell was controlled by addition of various amounts of TEOS. During the stirring of the reacting dispersion solution, another addition of TEOS was followed (ensure the uniformity of SiO_2 shell's surface). Synthesis of Au-NRs@SiO_2 with $<$4 nm thick SiO_2 shell requires three times

of addition of 10 µL TEOS aqueous solution, with 30 minutes interval for each addition. The solution was then allowed to react overnight with stirring. The Au-NRs@SiO_2 NPs were collected by centrifugation at 4,000 rpm for 5 minutes and re-dissolved in ethanol.

The sizes of the Au NRs and as well as the thickness of the silica shell were characterized by transmission electron microscopy (TEM). TEM images were obtained with a Tecnai G2 F30 microscope operated at an acceleration voltage of 120 kV.

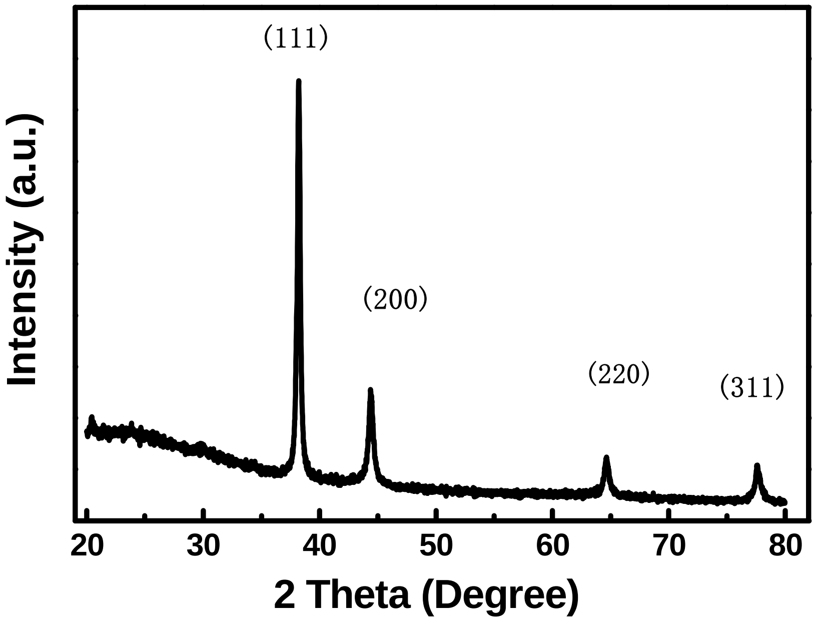

Figure S1. XRD spectrum of Au-NRs@SiO2 core/shell particles.

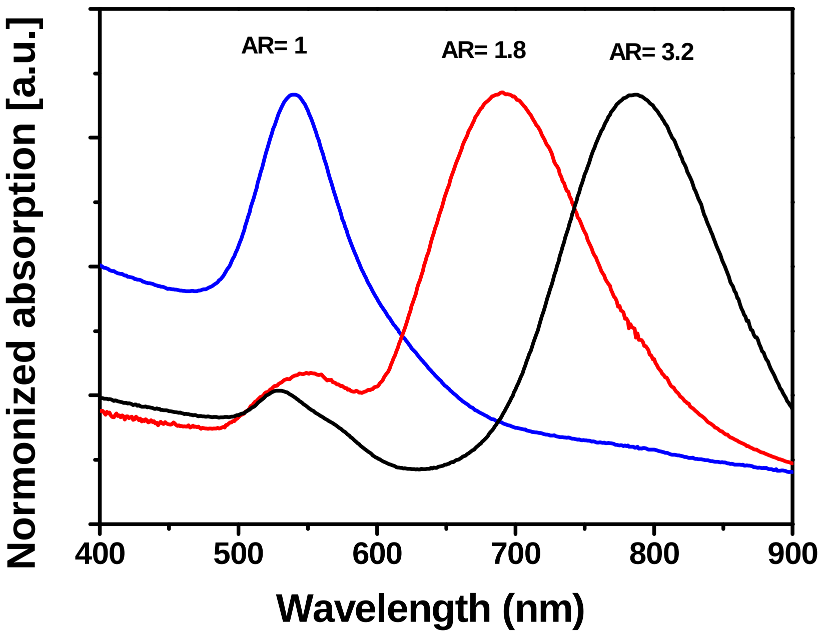

Figure S2. Absorption characterizations of prepared Au-NRs@SiO_2 NPs in ethanol.

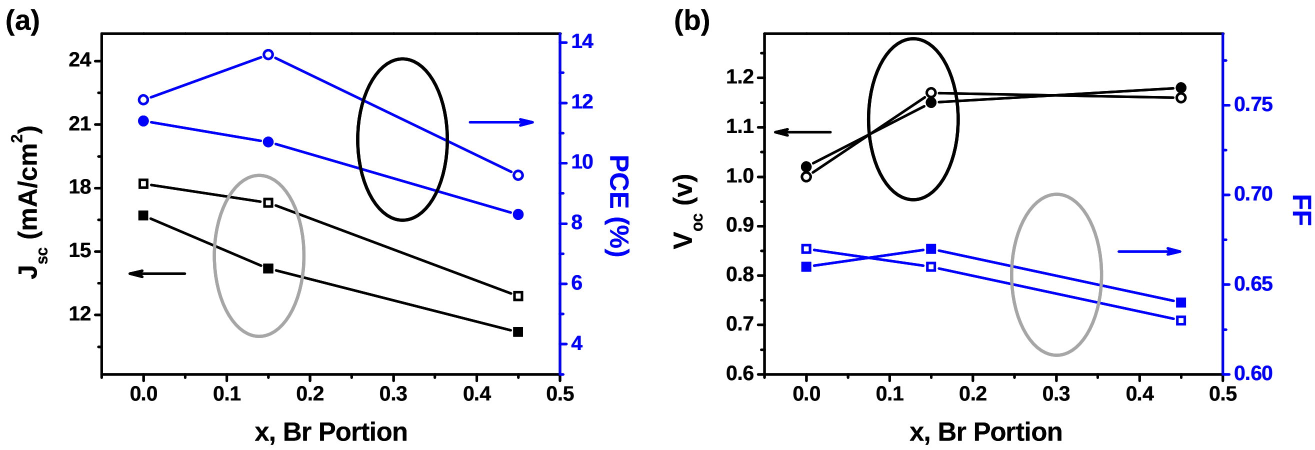

Figure S3. Variation of PCE and J_{sc} a), and V_{oc} and FF b) of PVSC devices with compact-NiO/meso-Al_2O_3/$CH_3NH_3PbI_{3-x}Br_x$/PCBM/BCP/Au configuration, with (open) and without Au@SiO_2 NRs (filled), where x = 0, 0.15, and 0.45.

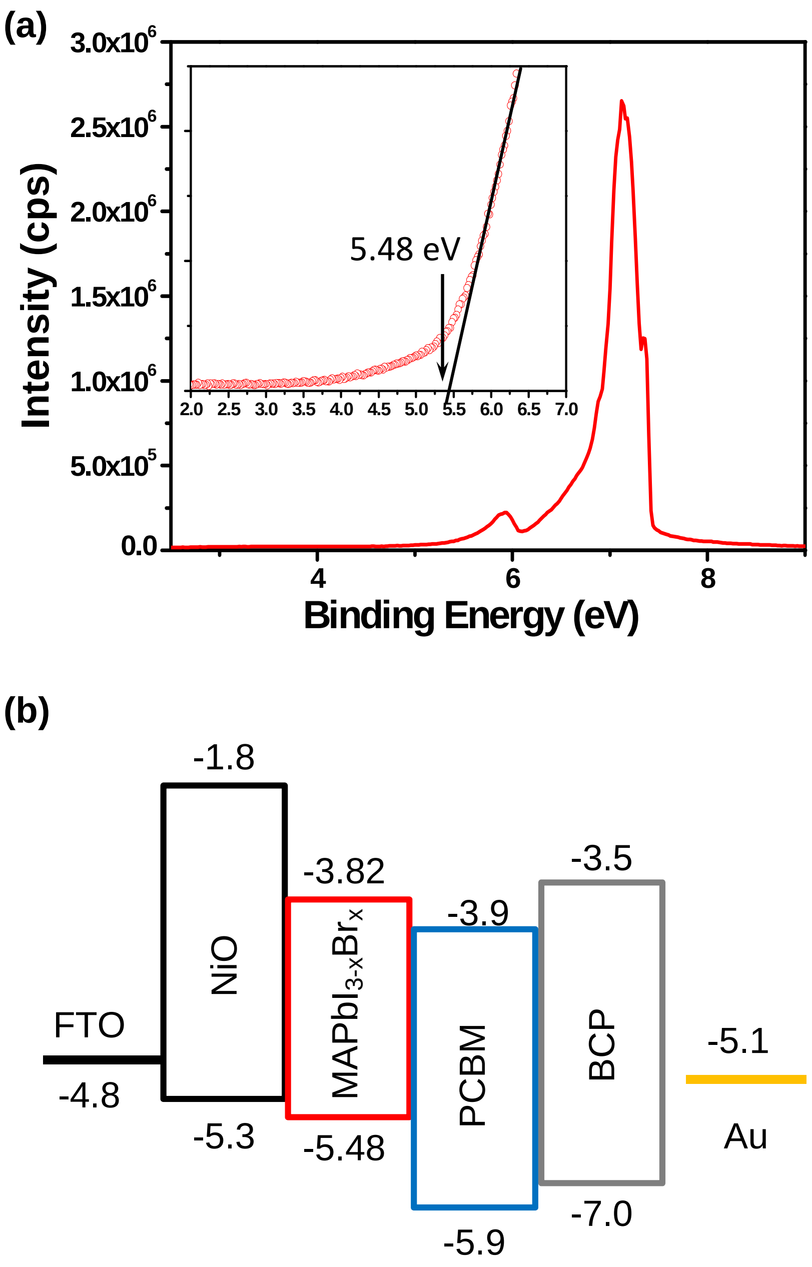

Figure S4. (a) Results of work function analysis by photoelectron spectroscopy for the two-step spin-coated $MAPbI_{3-x}Br_x$ perovskite film on glass substrate. And (b), diagram of the cell energy levels.

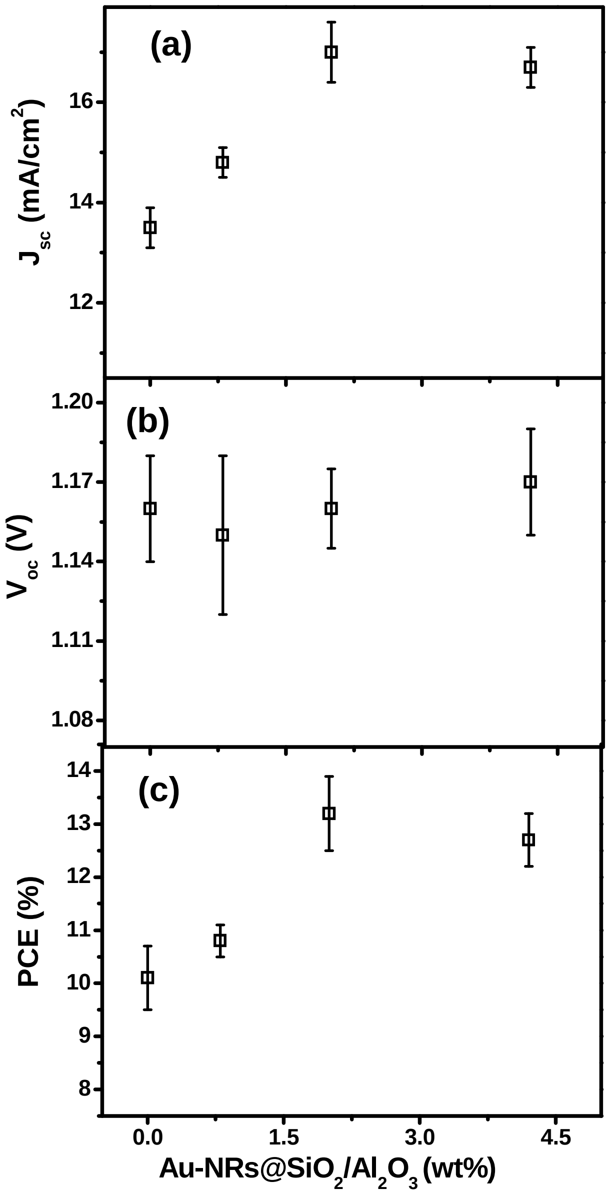

Figure S5. Concentration dependence of Au-NRs@SiO_2 NPs on the $CH_3NH_3PbI_{2.85}B_{0.15}$-based device performance: a) short circuit photocurrent,, b) open circuit voltage, and c) power conversion efficiency.

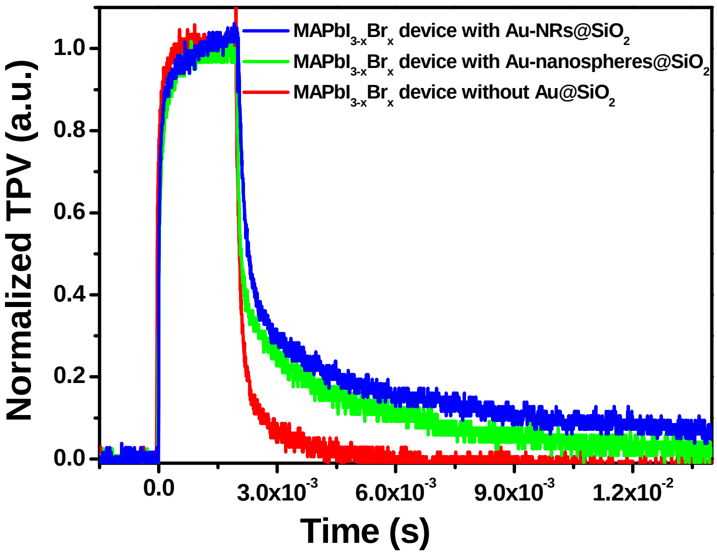

Figure S6. Transient photovoltage decay data obtained under 0.5 % sun illumination.

REFERENCES

[1] B. Nikoobakht, M. A. El-Sayed, *Chem. Mater.* **2003**, *15*, 1957;

[2] Z. Guo, C. Gu, X. Fan, Z. Bian, H. Wu, D. Yang, N. Gu, J. Zhang, *Nanoscale Res. Lett.* **2009**, *4*, 1428;
